# The neural mobilization technique modulates the expression of endogenous opioids in the periaqueductal gray and improves muscle strength and mobility in rats with neuropathic pain

**DOI:** 10.1186/1744-9081-10-19

**Published:** 2014-05-13

**Authors:** Fabio Martinez Santos, Leandro Henrique Grecco, Marcelo Gomes Pereira, Mara Evany Oliveira, Priscila Abreu Rocha, Joyce Teixeira Silva, Daniel Oliveira Martins, Elen Haruka Miyabara, Marucia Chacur

**Affiliations:** 1 Department of Anatomy, Laboratory of Functional Neuroanatomy of Pain, Institute of Biomedical Sciences, University of São Paulo, Av. Prof. Lineu Prestes, 2415, São Paulo 05508-000 SP, Brazil; 2 Special Laboratory of Pain and Signaling, Butantan Institute, University of São Paulo, Av. Vital Brasil, 1500, Butantã 05503-900 SP, Brazil; 3 Department of Anatomy, Laboratory of Skeletal Muscle Plasticity, Institute of Biomedical Sciences, University of São Paulo, Av. Prof. Lineu Prestes, 2415, 05508-000 São Paulo, SP, Brazil; 4 Department of Health Sciences, University Nove de Julho, São Paulo, SP, Brazil

**Keywords:** PAG, Mid-Brain, SFI, Neuropathic pain, Muscle, Opioids

## Abstract

**Background:**

The neural mobilization (NM) technique is a noninvasive method that has been proven to be clinically effective in reducing pain; however, the molecular mechanisms involved remain poorly understood. The aim of this study was to analyze whether NM alters the expression of the mu-opioid receptor (MOR), the delta-opioid receptor (DOR) and the Kappa-opioid receptor (KOR) in the periaqueductal gray (PAG) and improves locomotion and muscle force after chronic constriction injury (CCI) in rats.

**Methods:**

The CCI was imposed on adult male rats followed by 10 sessions of NM every other day, starting 14 days after the CCI injury. At the end of the sessions, the PAG was analyzed using Western blot assays for opioid receptors. Locomotion was analyzed by the Sciatic functional index (SFI), and muscle force was analyzed by the BIOPAC system.

**Results:**

An improvement in locomotion was observed in animals treated with NM compared with injured animals. Animals treated with NM showed an increase in maximal tetanic force of the tibialis anterior muscle of 172% (p < 0.001) compared with the CCI group. We also observed a decrease of 53% (p < 0.001) and 23% (p < 0.05) in DOR and KOR levels, respectively, after CCI injury compared to those from naive animals and an increase of 17% (p < 0.05) in KOR expression only after NM treatment compared to naive animals. There were no significant changes in MOR expression in the PAG.

**Conclusion:**

These data provide evidence that a non-pharmacological NM technique facilitates pain relief by endogenous analgesic modulation.

## Background

Injury to the peripheral nervous system (PNS) and the spinal cord contributes to the development of peripheral neuropathy; this damage then triggers ectopic focus in peripheral nerve fibers, which maintain continuous impulses to the central nervous system (CNS). These events, which are associated with central sensitization in the presence of peripheral nerve injury, contribute to the development of neuropathic pain [1,2]. Thus, injuries in any part of the course of this nerve can cause changes in sensory and motor structures involved in the area of innervation [3]. Studies have demonstrated that sciatic nerve injury in an animal model that is induced by chronic constriction injury (CCI) causes autonomic and motor changes, such as dystonic posturing and changes in locomotion [4]. It has been extensively reported that neuropathic pain is difficult to treat in clinical practice due to poor understanding of the cellular and molecular mechanisms involved in the development and maintenance of this type of pain [5,6]. Evidence suggests the involvement of opioid supraspinal regions in rats with neuropathic pain induced by CCI [7].

Opioids are potent analgesics that exert pharmacological and physiological effects through interactions with receptors distributed in several regions. Three subtypes of opioid receptors are known: MOR (μ), DOR (δ) and KOR (k). These subtypes are widely distributed in various systems, such as the immune, pituitary-adrenal axis, peripheral and central nervous systems, and they are important regulators of pain and inflammation [8,9]. Opioid peptides and their receptors are involved in a multitude of functions in the CNS, including stress, anxiety and antinociception [10]. In the peripheral nervous system, the receptors MOR, DOR and KOR are also found in the posterior root ganglion [11,12]. The first clues regarding the role of these receptors in peripheral analgesia appeared in 1987; the activation of opioid receptors were shown to reduce hyperalgesia, suggesting a decrease in spontaneous discharge of the fibers from C-type primary afferent neurons [13-15]. Between 1970–1980, it was hypothesized that the involvement of brain regions in the modulation of nociceptive primary afferent inputs originated from the peripheral nervous system [16]. Evidence indicated that electrical stimulation of the periaqueductal gray (PAG) induces intense analgesia in rats [16]. The PAG anatomically comprises a number of columns oriented in a longitudinal neuronal rostrocaudal direction, for example, the column dorsomedial (dm), the dorsolateral column (dl), the column lateral (Lat), the medial column (m) and the ventrolateral pillar (VL) [17]. The column of the ventrolateral PAG was thought to be the main region responsible for the release of endogenous opioids [18]; this region was examined to understand the relationship of the descending pain inhibitory system (endogenous opioids) on primary afferent nociceptive C fibers [19]. In addition, the PAG is responsible for the projection of neuron groups in the region of the rostral ventromedial medulla (RVM), where the nuclei raphe magnus, gigantocellular reticular nucleus and lateral gigantocellular are found [20]. Thus, the retransmission pulses coming from the PAG to the RVM are relayed to the spinal cord via the dorsolateral funiculus (LDF) causing attenuation of primary nociceptive stimuli by activating OFF cells (GABAergic) and inhibiting ON cells [21]; this is also the location where opioids and substance P play a fundamental role in the direct and indirect control over the descending modulation of pain [22].

The neural mobilization (NM) technique is a noninvasive physiotherapy method that has been proven to be clinically effective in reducing pain sensitivity after neuropathic pain [23]. Furthermore, Lopes et al. [24] demonstrated in humans that the NM technique can cause increased muscle strength of the quadriceps muscle that may be associated with improved axoplasmic flow. Furthermore, when applied in rats with neuropathic pain, the NM technique can regenerate the sciatic nerve by increasing the expression of zero nerve growth factor [25].

The current study tests the hypothesis that the NM technique reverses neuropathic pain by enhancing endogenous opioid-mediated pain modulatory systems. This hypothesis was evaluated by behavioral tests and Western blotting assays of the periaqueductal gray of adult neuropathic rats before and after treatment with NM.

## Methods

### Animals

Male Wistar rats, weighing between 180 and 220 g (approximately 2 months old), were used in all experiments. The rats were singly housed and maintained on a 12:12 h light/dark cycle and were adapted to the experimental environment three days before the experiments started. All animals were tested during the light cycle at the same time of the day (9:00 a.m. – 12:00 p.m.). All procedures were approved by the Institutional Animal Care Committee of the University of São Paulo (protocol number 091 – book number 02/2012) and performed in accordance with the guidelines for the ethical use of conscious animals in pain research published by the International Association for the Study of Pain [26].

### Surgical procedure

#### Chronic constriction injury - (CCI)

For the induction of neuropathic pain, chronic constriction of the sciatic nerve was performed as previously described by Bennett and Xie in [4]. In short, rats were anesthetized with halothane (Cristalia, Brazil) [4]. The common sciatic nerve was exposed at the level of the middle of the thigh by blunt dissection through the biceps femoris. Proximal to the sciatic trifurcation (about 7 mm), the nerve was freed of adhering tissue, and 4 ligatures (4.0 chromic gut) were tied loosely around it with approximately 1 mm spacing. Great care was taken to tie the ligatures such that the diameter of the nerve was seen to be just barely constricted. The incision was closed in layers. In sham-operated rats, the same incision was made, as described above, the sciatic nerve was exposed at the same level but left unaffected, and the muscle was sutured. This animals was used as a control. Each rat was closely observed during the recovery from anesthesia and then returned to the home cage and carefully observed during the following 24 hours. During the 5 day-period after CCI, walking and cage exploration, the degree of limping, and the conditions of the hindpaw, including signs of excessive grooming or autotomy, were all closely observed.

#### Sciatic functional index (SFI)

The SFI was then measured under normal conditions, 14 days after nerve injury, as well as the third, seventh and tenth sessions of the neural mobilization and sham procedures. We used a total of 5 animals for each group. To evaluate the functional activity, the sciatic functional index, which evaluates the footprints of animals, was used [27]. The footprints of the animals were obtained by the method proposed by De Medinaceli et al. [27,28] using paper strips impregnated with blue bromofenols. A walkway (44 cm × 8.7 cm) was constructed in wood according to De Medinaceli et al. [27,28] and closed on the sides; a hutch was provided at the end where the animal could find shelter and food pellets after traveling throughout its length [28,29]. The animals were trained to walk on the runway prior to the initial steps and were considered fit if they crossed the footbridge (e.g., the initial opening to the hutch) without hesitation. Once trained, the initial steps were performed with the animals walking with their hind legs moistened with water on the dyed leaves. Based on the footprints obtained, the following parameters were measured (using a MARK – MYTUTOYO caliper): (1) print length, (2) spread of the toes, and (3) intermediate toes. These data were collected on the normal side (non-operated) (NPL, NTS and NIT) and the experimental side (operated) (EPL, ETS and EIT). The data were analyzed using the equation developed by De Medinaceli et al. [27] and adapted by Bain et al. (1989) and Hare et al. (1992): SFI = - 38.3 × [(EPL - NPL)/NPL] + 109.5 × [(ETS - NTS)/NTS] + 13.3 × [(EIT - NIT)/NIT] - 8.8. Where: N = normal; E = Experimental; PL = print length; TS = spread of toes; IT = intermediate toes [30,31]. The results obtained from this equation are expressed in a negative percentage of normal function, where 0 (zero) (standard deviation of -11 to 11) corresponds to normal function or no disability, and -100 (minus one hundred) correspond to full dysfunction.

### In vivo muscle function experiments

Based on Pereira et al. [32] rats were anesthetized with tribromoethanol (20 mg/100 g BW, i.p.). The exposition of their sciatic nerves was performed through a lateral incision on the thigh, and an electrode was coupled. The innervations of the sciatic nerve to the tibialis anterior muscle were cautiously isolated from those originated from other nerves. Then, the animals were placed on an acrylic platform with a metallic bar crossing their knees to fix their limbs. The tibialis anterior tendon was connected to a force transducer coupled to a computer that was used to assemble and analyze force generated by the muscle contraction [32]. Muscle twitch and tetanic forces were documented using the Biopac Systems (Goleta©, CA, USA). Muscle strength was analyzed using the software AcqKnowledge 3.9.1.6. Rats were submitted to external warming in order to maintain their core temperature throughout the procedure.

At the start of the experiment, the muscle was set to the optimum length (*L*_*0*_, defined as a length resulting in maximum twitch force) and a two-minute rest period was applied between stimuli (data not shown). In order to achieve the maximal plateau force with minimal frequency, we used 200Hz stimuli for measuring the maximal tetanic force.

Based on Chan and Head (2010), isolated twitches (0.2Hz) were generated over a 2-min period, followed by a maximum tetanic contraction (induced for 2-s) in each muscle (at 200Hz) [33]. We observed no differences in terms of twitch parameters, such as the time-to-peak and half-relaxation time (data not shown). The results were expressed as maximum tetanic force.

### Neural mobilization (NM) technique

The NM technique used here has been described by Butler (1989) and adapted by our laboratory [23,34]. Briefly, rats were anesthetized with isoflurane, with a continuous flow of medicinal oxygen throughout the procedure (5 ml/L – 0, 5%/L). After anesthesia, the animals were positioned in the left lateral position to mobilize the right side (ipsilateral to the CCI). The right knee joint was then positioned in full extension (at 0 degrees) and remained so throughout the session. Moreover, the right hip joint was bent between 70 to 80 degrees with the knee in extension until a small resistance was induced by stretching the muscles from the compartimentum posterius femoris (biceps m., femoris m., semimembranosus m., and semitendinosus m.). After the manipulator felt the resistance, the angle of the joint was interrupted. At this time, the ankle joint was angled between 30 to 45 degrees, using the same principle described above. After all of the joints were positioned with minimal resistance of those muscles, oscillatory movements were initiated. The right ankle joint was manipulated in dorsi-flexion (30 to 45 degrees) at approximately 20 oscillations per minute for 2 minutes, followed by a 25-second pause for rest. The treatment was performed for ten minutes; in the last minute, the cervical spine was fully flexed with the purpose of tensioning the entire neuraxis [35]. Treatment with the NM technique started 14 days after the injury or sham procedure, and the NM sessions were conducted every other day for a total of 10 sessions. The animals was divided in 5 groups, naive, CCI, CCINM, Sham and ShamNM, we used a total of 5 animal in each group, after the last NM sessions, animals were sacrificed for immunoblotting and in vivo muscle experiments as described below and above respectively.

#### Immunoblotting

Western blotting analyses were performed on samples from individual animals. Neuropathic (CCI), sham and naive rats were sacrificed by decapitation under isofluorane anesthesia, and the PAG was quickly removed and homogenized in an extraction buffer containing 100 mM Tris, pH 7.4, 10 mM EDTA, 2 mM PMSF, and 10 μg/mL aprotinin. After extraction, the homogenates were centrifuged at 11,500 × g for 20 min, and the protein concentration of the supernatant was determined using the Bradford protein assay with albumin as a standard (Bio-Rad, USA) [36]. Samples containing 75 μg protein were loaded on a 12% acrylamide gel and electrotransferred to nitrocellulose membranes using a Bio-Rad miniature transfer apparatus for 1.5 h at 120 V. After transfer, the membranes were treated for 2 h at room temperature with a blocking solution containing 5% powdered milk, washed and incubated overnight at 4°C with rat monoclonal antibodies against Anti-MOR or (μ), DOR or (δ) or KOR or (k) (all 1:250 – Santa Cruz Biotechnology, INC) [37-39]. Membranes were then washed and incubated for 2 h at room temperature with a peroxidase-conjugated, goat anti-rabbit secondary antibody (to MOR and DOR) diluted 1:5000 (Jackson ImunuResearch, Laboratories, INC) and a donkey anti-goat (to KOR) secondary antibody diluted 1:5000 (Jackson ImunuResearch, Laboratories, INC). In all immunoblotting experiments, β-actin (mouse, 1:15000; Sigma-Aldrich, USA) was used as an internal control. The specific antibody bind was visualized using a chemoluminescence kit (Amersham Biosciences). The blot was densitometrically analyzed using NIH-Scion Image 4.0.2 (Scion Corporation, USA).

### Statistical analysis

Results are presented as the mean ± SEM. The data were analyzed using GraphPAD Prism, version 4.02 (Graph-Pad Software Inc., San Diego, CA). Statistical comparison of more than 2 groups was performed using analysis of variance (ANOVA); differences between means were determined by Tukey's multiple comparison test when appropriate. In all cases, *p* < 0.05 was considered statistically significant [40].

## Results

### Assessment of locomotor function through the sciatic functional index (SFI)

**Figure 1:**
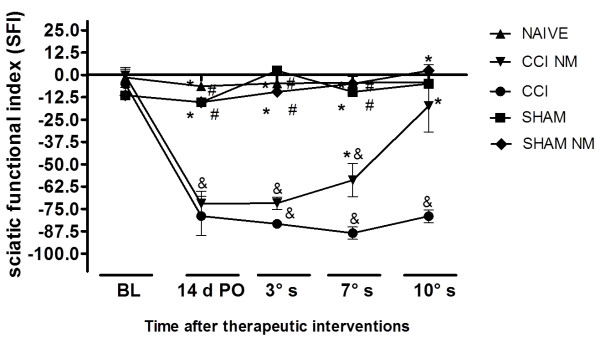
**Analysis of Sciatic Functional Index (SFI).** Motor dysfunction was induced by chronic constriction injury (CCI) to the sciatic nerve. Functional assessment was evaluated, 14 days after injury (14d PO), after the third (3°s); seventh (7°s); and tenth (10°s) session (s) using the SFI. The results obtained are expressed as a percentage of normal function, where 0 (zero) corresponds to normal function or no disability and -100 (minus one hundred) corresponds to total dysfunction. The results are expressed as the mean ± S.E.M. Five animals per group. *p <0.05 per CCI comparison group. #p <0.05 compared to the CCI NM group and p <0.05 compared to the initial measurement.

Initially, all animals were tested for the sciatic functional index to evaluate the response of locomotion and to obtain the initial measures (BL - baseline). On the fourteenth day after injury, all animals were re-evaluated to check for possible changes in the SFI. In this period, the animals in groups CCI and CCI NM partially unloaded body weight on the right leg (injured), reflecting in complete adduction of their fingers and sagging. These changes were represented by a decrease in SFI values compared with baseline measures and control groups: Sham (Sham and Sham NM) and naive groups showed no changes in SFI values. It is worth mentioning that the observed decrease in the SFI in the CCI group remained during all evaluation measures of locomotion until the date of euthanasia (Figure 1). It is essential to highlight that the dysfunction in ambulation was characterized by damage in the sciatic nerve (CCI).When the animals from the CCI NM group were submitted to the SFI tests 14 days after surgery, the same signal from the CCI group was observed (e.g., partial discharge of body weight on the right leg (op) reflecting incomplete adduction of the fingers and sagging). However, during treatment with NM, the animals showed an increase to values close to zero at the SFI, representing an improvement of locomotion. Both the Sham (Sham and Sham NM) and naive control groups remained at values close to the initial measures without significant changes (Figure 1). The changes observed between groups CCI and CCI NM reflect the improvement caused by the NM technique, demonstrating a beneficial effect of this technique on motor dysfunction caused by chronic constriction injury of the sciatic nerve.

### In vivo muscle function experiments

**Figure 2:**
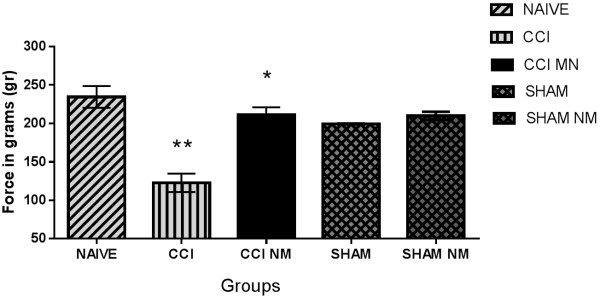
**Analysis of *in vivo *muscle function experiments.** The results are expressed in grams. The results are expressed as the mean ± S.E.M. n = 5. **p <0.001 comparing the CCI and control groups (naive, sham and sham NM) *p <0.001 comparing the CCI NM and CCI groups.

Our results showed that the maximal tetanic force of the tibialis anterior muscle from the CCI group decreased by 52% (p < 0,001) compared to the naive group. However, muscles from the CCI NM group showed an increase of maximal tetanic force of 172% (p < 0,001) compared to the CCI group and a decrease of 10% compared to the naive group (Figure 2). No difference in maximal tetanic contraction was observed between the Sham groups and the naive group.

### Effects of NM on the expression of opioid receptors in the periaqueductal gray

We employed analysis of the DOR, MOR and KOR protein expression levels to evaluate how CCI and NM treatment can alter opioid protein levels.

**Figure 3:**
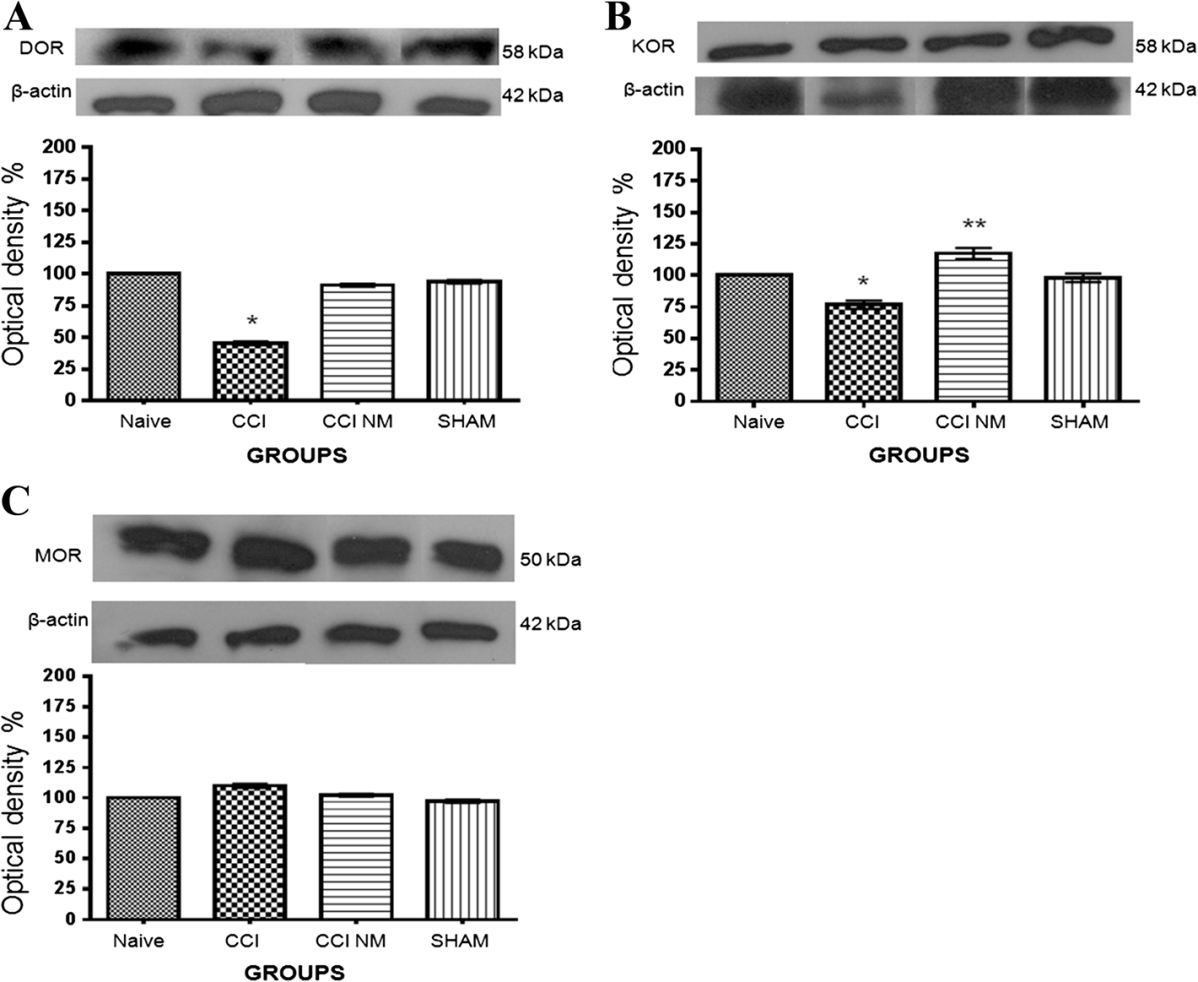
**Densitometry analysis of DOR (A), KOR (B) and MOR (C) expression in the periaqueductal gray after CCI injury.** The normalized average between sham and experimental groups (CCI) is reported. Values measured for naive animals were considered 100%. Data are reported as the mean ± SEM of 6 animals per group. **A)** *p < 0.001 comparing the CCI group with the naive group. **B)** *p < 0.05 the CCI group with naive animals, **p < 0.001 comparing the CCI NM group with the other groups.

Our results showed a decrease of 53% (*p* < 0.001) in DOR levels after CCI injury compared to those from naive animals and a return to basal levels after NM treatment. The difference between the CCI NM and CCI groups was approximately 50% (*p* < 0.001) (Figure 3A).

Regarding KOR protein expression, we observed a decrease of 23% (*p* < 0.05) in KOR levels after CCI injury compared to naive animals and an increase of 17% in KOR expression after NM treatment (p < 0,05). The difference between the CCI NM and CCI groups was approximately 40% (*p* < 0.01) (Figure 3B).No differences in MOR expression between the groups were observed when the periaqueductal gray was analyzed (Figure 3C).No difference was observed between the naive and sham-NM groups or between the sham and sham-NM groups (data not show). No difference was observed in the β-actin expression between control and experimental sides at all time points tested (Figure 3).

## Discussion

The NM technique reversed the deficit in muscle strength and locomotor dysfunction observed in the CCI model of induced neuropathic pain. Moreover, this non-pharmacological therapy increased the expression of opioid receptors, especially the DOR and KOR in the PAG, suggesting that these effects may occur through activation of endogenous opioid-mediated pain modulatory systems.

Regarding the lack of MOR opioid receptors be involved in our model is intriguing. Different studies showed distinct results regarding different type of opioid receptor. Konno et al. [41] showed the involvement of KOR opioid receptors in the antinociceptive effect induced by a peptide in the nociceptive effect by prostaglandin E_2_ and carrageenin [41]. In addition, other studies also demonstrated difference in opioid expression; studies showed an involvement of only KOR and DOR opioids receptors in a cancer pain model [42] and in a crude crotalic venom model [43], both authors do not observed the involvement of MOR opioid receptors in their models.

Also, studies showed that opioid receptor expression is distinctly regulated by the presence of acute or chronic injury, as prostaglandin E2 increases the expression and activity (by the selective agonists) of MOR and KOR-opioid receptors in rat DRG and nerve paw and decreases the expression and activity of DOR-opioid receptors. In contrast, chronic constriction injury of sciatic nerve up-regulates DOR-opioid receptors [44].

Opioids receptors are expressed in brain regions that modulate nociception, including anterior cingulated cortex, thalamus and periaqueductal grey (PAG) [45]. These areas have anatomical connections and involvement in establishment chronic pain states [7].

This hypothesis was confirmed by previous works demonstrating that analgesia by noninvasive techniques is mediated via neuronal mechanisms correlated with the central nervous system, with endogenous opiates and descending inhibitory mechanisms being the most recognized [46-49]. Furthermore, the importance of the PAG in the inhibitory system is well characterized in studies with the administration of DAMGO (a mu-opioid agonist) and DPDPE (a delta-opioid agonist), which were able to attenuate neuropathic pain symptoms in rats; in contrast, naloxone (opioid antagonist, administered i.p.) reversed these effects [49].

Our results confirm and extend previous findings that locomotion and muscle strength is impaired in the neuropathic pain syndrome [50-53] observed in the CCI model 14 days post injury. Our results demonstrated that ten NM sessions restored locomotor dysfunction and muscle strength associated with the CCI condition. Medinaceli et al. [27] were the first researchers to describe the sciatic functional index as a tool to assess the locomotor dysfunction followed by a sciatic nerve lesion and Wallerian degeneration [27]. Regarding locomotor dysfunction, our data demonstrate the effects of NM in improving motor dysfunction induced by a CCI injury. In addition, the same group of animals increased maximal tetanic force when compared to animals without NM treatment. Our data corroborate studies showing that nerve crush injury induces locomotor dysfunction due to degeneration of axons [54-57] and that CCI injury causes Wallerian degeneration ADDIN EN.CITE [58-60]. Interestingly, previous studies from our group demonstrated an increase in myelination and zero protein levels in the sciatic nerve of CCI rats submitted NM treatment, demonstrating the regenerative efficiency of this technique [25]. This finding suggests, once again, that NM it is a good manual technique to improve pain behavior with neuropathic pain injury.

Because the literature remains scarce with regard to scientific studies investigating why and how rehabilitation techniques are able to produce an improvement in the patient’s condition, we have demonstrated the relationship between neural mobilization and opioids for the first time; this procedure has been widely used to treat clinical syndromes, such as nerve compression, radiculopathy and postsurgical neuropathies [23,61,62].

In summary, the NM technique reverses the deficit in muscle strength and locomotor dysfunction observed in an animal model of neuropathic pain. Non-pharmacological treatment also increases opioid receptor levels in an area of the brainstem known to be important in pain modulation. These data provide evidence that NM facilitates pain relief through endogenous analgesic modulation. Clinical studies in humans have shown that NM reduces neuropathic pain; however, the molecular mechanisms remain elusive [61-64]. The current findings have significant implications in the clinical rehabilitation of patients with neuropathic pain.

Despite the efficacy and long-lasting antinociceptive effect observed in our studies, the possibilities that neural mobilization may represent an important therapeutic approach to control chronic pain, should be carefully evaluated through future studies.

## Competing interest

The authors declare that they have no competing interest.

## Authors’ contribution

SFM carried out surgery, behavioral experiments, neural mobilization (NM) technique and drafted the manuscript; GLH carried out Sciatic functional index; PMG carried out In vivo muscle function experiments; OME carried out behavioral experiments; RPA carried out statistical analyses; SJT carried out behavioral experiments; MDO carried out Immunoblotting and drafted the manuscript; MEH carried out In vivo muscle function experiments; CM drafted the manuscript and revised all experiments conducted by the students. All authors read and approved the final manuscript.
